# Overall survival results from the randomized phase 2 study of palbociclib in combination with letrozole versus letrozole alone for first-line treatment of ER+/HER2− advanced breast cancer (PALOMA-1, TRIO-18)

**DOI:** 10.1007/s10549-020-05755-7

**Published:** 2020-07-18

**Authors:** Richard S. Finn, Katalin Boer, Igor Bondarenko, Ravindranath Patel, Tamas Pinter, Marcus Schmidt, Yaroslav V. Shparyk, Anu Thummala, Nataliia Voitko, Eustratios Bananis, Lynn McRoy, Keith Wilner, Xin Huang, Sindy Kim, Dennis J. Slamon, Johannes Ettl

**Affiliations:** 1 David Geffen School of Medicine, University of California Los Angeles, 2825 Santa Monica Blvd, Suite 200, Santa Monica, CA USA; 2 Onkologia, Szent Margit Korhaz, Budapest, Hungary; 3 Dnipropetrovsk State Medical Academy, Dnipropetrovsk, Ukraine; 4 Comprehensive Blood and Cancer Center, Bakersfield, CA USA; 5 Petz Aladar Megyei Oktato Korhaz, Gyor, Hungary; 6 Department of Obstetrics and Gynecology, University Medical Center Mainz, Mainz, Germany; 7 Lviv State Oncologic Regional Treatment and Diagnostic Center, Lviv, Ukraine; 8 Comprehensive Cancer Centers of Nevada, Las Vegas, NV USA; 9 Kyiv City Clinical Oncology Center, Kyiv, Ukraine; 10 Pfizer Inc, New York, NY USA; 11 Pfizer Inc, San Diego, CA USA; 12 Department of Obstetrics and Gynecology, Klinikum Rechts Der Isar, Technische Universität München, München, Germany

**Keywords:** Advanced breast cancer, ER+/HER2−, Letrozole, Overall survival, Palbociclib

## Abstract

**Purpose:**

Palbociclib is a cyclin-dependent kinase 4/6 (CDK4/6) inhibitor, approved in combination with endocrine therapy for the treatment of women and men with hormone receptor–positive, human epidermal growth factor receptor 2–negative advanced breast cancer (HR+/HER2− ABC). In the phase 2, open-label, PALOMA-1 trial, palbociclib plus letrozole significantly prolonged progression-free survival (PFS) versus letrozole alone (hazard ratio, 0.488; 95% CI 0.319‒0.748; *P* = 0.0004; median PFS, 20.2 vs 10.2 months, respectively) in postmenopausal women with estrogen receptor–positive (ER+)/HER2− ABC. Here, we present the final overall survival (OS) and updated safety results.

**Methods:**

Postmenopausal women with ER+/HER2− ABC were randomized 1:1 to receive either palbociclib (125 mg/day, 3/1 schedule) plus letrozole (2.5 mg/day, continuous) or letrozole alone (2.5 mg/day, continuous). The primary endpoint was investigator-assessed PFS; secondary endpoints included OS and safety.

**Results:**

A total of 165 patients were randomized. At the data cutoff date of December 30, 2016 (median duration of follow-up, 64.7 months), the stratified hazard ratio for OS was 0.897 (95% CI 0.623–1.294; *P* = 0.281); median OS in the palbociclib plus letrozole and letrozole alone arms was 37.5 and 34.5 months, respectively. The median time from randomization to first subsequent chemotherapy use was longer with palbociclib plus letrozole than letrozole alone (26.7 and 17.7 months, respectively). The most frequently reported adverse event in the palbociclib plus letrozole arm was neutropenia (any grade, 75%; grade 3 or 4, 59%).

**Conclusions:**

Palbociclib plus letrozole treatment led to a numerical but not statistically significant improvement in median OS.

Pfizer Inc (NCT00721409)

## Introduction

Breast cancer is the most common type of cancer in women worldwide [1]; approximately 70% of breast cancers are hormone receptor–positive/human epidermal growth factor receptor 2–negative (HR+/HER2−) [2]. Although the long-term prognosis is good for patients whose disease has not spread, historically, the 5-year survival rate for patients who develop or present with metastatic breast cancer is only approximately 25% [2, 3]. Single-agent endocrine therapy (ET; including antiestrogens and aromatase inhibitors [AIs]) had long been the mainstay of therapy for first-line treatment in postmenopausal women, with a better safety profile and quality of life compared with standard chemotherapy [4]. Recently, treatment with the cyclin-dependent kinase 4/6 (CDK4/6) inhibitor palbociclib in combination with endocrine therapy (ET) was incorporated in the National Comprehensive Cancer Network treatment guidelines for patients with HR+/HER2− advanced breast cancer (ABC) [5].

Palbociclib is a first-in-class, potent, highly selective, orally administered, reversible CDK4/6 inhibitor [6]. Preclinical studies revealed that palbociclib in combination with ET preferentially and synergistically inhibited the cell cycle in human estrogen receptor–positive (ER+) breast cancer cell lines [7]. Based on these preclinical data, the phase 2 PALOMA-1 clinical trial was initiated to investigate the efficacy and safety of palbociclib plus ET as first-line treatment for postmenopausal women with ER+/HER2− ABC [8]. Progression-free survival (PFS) was the primary endpoint for this study. At the time of final analysis for PFS (data cutoff date, November 29, 2013), PFS was significantly prolonged in the palbociclib plus letrozole arm compared with the letrozole alone arm (hazard ratio, 0.488; 95% CI 0.319–0.748; 1-sided *P* = 0.0004; median PFS, 20.2 vs 10.2 months, respectively) [8]. The results from this study led to the accelerated US Food and Drug Administration approval of palbociclib in combination with letrozole for the treatment of ER+/HER2− ABC in February 2015.

At the time of final PFS analysis, an interim overall survival (OS) analysis was performed. The hazard ratio was 0.813 (95% CI 0.492–1.345; 2-sided *P* = 0.42) [6]. A longer median OS was observed in the palbociclib plus letrozole arm compared with the letrozole alone arm (37.5 vs 33.3 months, respectively) [8]. Of note, the PALOMA-1 trial was not designed to perform formal hypothesis testing for OS due to the relatively small sample size; at the time of final PFS analysis, 30 (36%) and 31 (38%) OS events had occurred in the palbociclib plus letrozole and letrozole alone arms, respectively [8].

The phase 3 PALOMA-2 study confirmed the PFS benefit observed in PALOMA-1; PFS was significantly longer in the palbociclib plus letrozole arm compared with the placebo plus letrozole arm as first-line treatment in postmenopausal women with ER+/HER2− ABC (hazard ratio, 0.563; 95% CI 0.461–0.687; *P* < 0.0001; median PFS, 27.6 vs 14.5 months, respectively) [9]. In the phase 3 PALOMA-3 trial, PFS was also significantly longer in the palbociclib plus fulvestrant arm compared with the placebo plus fulvestrant arm in women with HR+/HER2− ABC, regardless of menopausal status, whose disease had progressed on prior ET, in either the adjuvant or metastatic setting (hazard ratio, 0.497; 95% CI 0.398–0.620; *P* < 0.0001; median PFS, 11.2 vs 4.6 months, respectively) [10, 11]. PALOMA-3 was the first phase 3 study of a CDK4/6 inhibitor to report OS results. The final OS analysis from PALOMA-3 showed longer median OS in the palbociclib plus fulvestrant arm compared with the placebo plus fulvestrant arm; however, this difference was not statistically significant (stratified hazard ratio, 0.81; 95% CI 0.64–1.03; 2-sided *P* = 0.09; median OS, 34.9 vs 28.0 months, respectively) [10]. Here, we report the final OS and updated safety results from PALOMA-1, the first randomized study of a CDK4/6 inhibitor in ABC with the longest follow-up to date.

## Methods

### Study design and patients

Detailed methods for the phase 2 PALOMA-1 clinical trial have been previously published [8]. PALOMA-1 (NCT00721409) was an international, phase 2, open-label, multicenter, randomized clinical trial that enrolled postmenopausal women with ER+/HER2− ABC. Patients were enrolled into 2 cohorts that accrued sequentially. Patients were enrolled into cohort 1 based on ER+/HER2− status alone, whereas cohort 2 enrolled patients with tumors with cyclin D1 amplification, loss of p16, or both. After an interim analysis, accrual into cohort 2 was stopped, and the analysis plan was amended to combine both cohorts for the analyses of the study endpoints.

For both cohorts, randomization was stratified by disease site (visceral, bone only, or other) and disease-free interval (DFI; > 12 months from the end of adjuvant therapy to recurrence versus ≤ 12 months from the end of adjuvant therapy to recurrence or de novo advanced disease). Key inclusion criteria were ER+/HER2− tumors, women, aged ≥ 18 years, postmenopausal status, Eastern Cooperative Oncology Group (ECOG) performance status 0 or 1, adequate organ function, and measurable disease per Response Evaluation Criteria in Solid Tumors (RECIST) criteria or bone-only disease. Key exclusion criteria were prior systemic treatment for advanced disease, prior treatment with (neo)adjuvant letrozole with disease recurrence ≤ 12 months, and prior treatment with a CDK inhibitor.

### Treatment

Patients were randomized 1:1 to receive either oral palbociclib 125 mg/day, 3 weeks on treatment followed by 1 week off (3/1 schedule) plus continuous oral letrozole 2.5 mg/day or continuous letrozole 2.5 mg/day alone. Study treatment continued until disease progression, unacceptable toxicity, study withdrawal, or death. Palbociclib dose modifications, including cycle delay, dosing interruption, and dose reduction, were permitted to manage adverse events (AEs). No letrozole dose adjustment was allowed, but dosing interruptions were permitted.

### Outcomes

The primary study endpoint was investigator-assessed PFS, defined as the time from randomization to the date of first documentation of objective progression (based on RECIST v.1.0) or death due to any cause. Secondary endpoints included OS, objective response, clinical benefit rate, duration of response, and safety.

### Statistical analysis

Overall survival was defined as the time from randomization date to the date of death due to any cause. OS was assessed in the intention-to-treat population, defined as all randomized patients, and in subgroups based on baseline demographic and disease characteristics using the Kaplan–Meier method; log-rank tests were used to compare OS between treatment arms. Cox regression models were used to estimate the treatment hazard ratio and associated 95% CI. The time to first use of subsequent chemotherapy was also assessed using the Kaplan–Meier method. Safety was evaluated in the as-treated population, defined as all patients treated with at least 1 dose of study treatment and reported as AEs graded based on the National Cancer Institute Common Terminology Criteria for Adverse Events v3.0.

## Results

### Patient population

In total, 84 and 81 patients were randomized into the palbociclib plus letrozole arm and letrozole alone arm, respectively. Patient demographics and baseline disease characteristics were relatively balanced between the treatment arms (Table 1). Median age was similar in both treatment arms, and approximately half of the patients in each treatment arm had visceral metastases. Approximately half of the patients in each treatment arm had received prior adjuvant systemic treatment, with the most common treatments in both treatment arms being chemotherapy (41% and 46% in the palbociclib plus letrozole and letrozole arms, respectively) and hormonal therapy (32% and 36% in the palbociclib plus letrozole and letrozole arms, respectively). As of the data cutoff date (December 30, 2016), 80 patients in the palbociclib plus letrozole arm and 79 patients in the letrozole arm had permanently discontinued from the study. The most frequent reason for permanent discontinuation from the study treatment was objective disease progression (63% and 77% of patients in the palbociclib plus letrozole and letrozole arms, respectively). Thirteen and 2 patients in the palbociclib plus letrozole and letrozole arms, respectively, permanently discontinued the study treatment due to AEs.

**Table 1:** Patient demographics and baseline disease characteristics (ITT population)

	PAL + LET (*n* = 84)	LET (*n* = 81)
Age, median (range), y	63 (41–89)	64 (38–84)
Age distribution, years, *n* (%)		
18‒44	2 (2)	4 (5)
45‒64	45 (54)	38 (47)
≥ 65	37 (44)	39 (48)
Race, *n* (%)		
White	76 (90)	72 (89)
Black	1 (1)	1 (1)
Asian/other	7 (8)	8 (10)
ECOG performance status, *n* (%)		
0	46 (55)	45 (56)
1	38 (45)	36 (44)
Disease stage, *n* (%)		
Stage III	3 (4)	1 (1)
Stage IV	81 (96)	80 (99)
Disease site, *n* (%)		
Visceral	38 (45)	43 (53)
Bone only	16 (19)	12 (15)
Other (nonvisceral)	30 (36)	26 (32)
Disease-free interval, *n* (%)		
> 12 months from adjuvant to recurrence	25 (30)	30 (37)
≤ 12 months from adjuvant to recurrence or de novo advanced disease	59 (70)	51 (63)
De novo advanced disease	44 (52)	37 (46)
Prior systemic treatment, *n* (%)		
None	44 (52)	37 (46)
Chemotherapy	34 (40)	37 (46)
Antihormonal	27 (32)	29 (36)
Tamoxifen	24 (29)	25 (31)
Anastrozole	8 (10)	12 (15)
Letrozole	2 (2)	1 (1)
Exemestane	4 (5)	2 (2)

*ECOG* Eastern Cooperative Oncology Group, *ITT* intention-to-treat, *LET* letrozole, *PAL* palbociclib

### Treatment exposure

As of the data cutoff date, the median duration of follow-up was 64.7 months (palbociclib plus letrozole arm, 69.3 months; letrozole arm, 59.0 months). The median duration of treatment was 13.8 months (range, 0.2‒77.0) in the palbociclib plus letrozole arm and 7.6 months (range, 0.9–64.7) in the letrozole arm. Mean relative dose intensity, defined as the actual dose divided by the intended dose, was 94% and 100% in the palbociclib plus letrozole and letrozole treatment arms, respectively. Thirty-four patients (41%) in the palbociclib plus letrozole arm had the palbociclib dose reduced to 100 mg/day, and 12 patients had the palbociclib dose further reduced to 75 mg/day. Dosing interruptions due to AEs occurred in 30 patients (36%), and cycle delays associated with AEs occurred in 38 patients (46%) in the palbociclib plus letrozole arm. In the letrozole arm, 3 patients (3.9%) had letrozole dosing interruption due to AEs.

### Efficacy

**Fig. 1:**
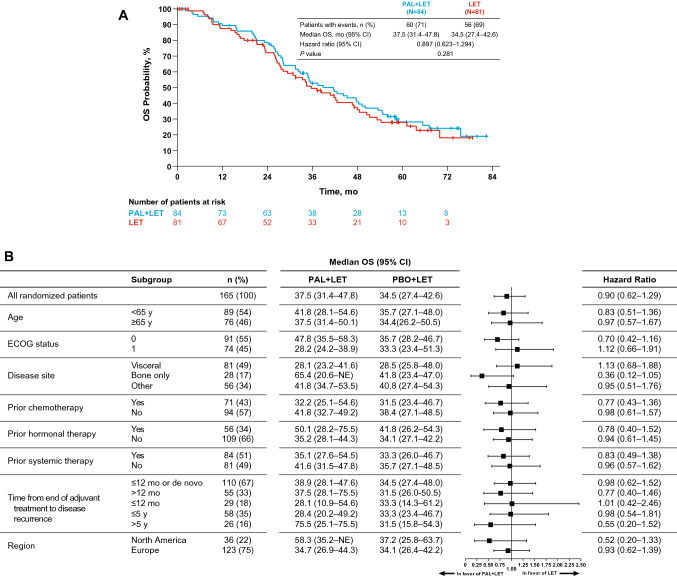
Overall survival in the ITT population and by subgroup. **a** Kaplan–Meier curve of OS in the ITT population. **b** Forest plot of OS by subgroup. *ECOG* Eastern Cooperative Oncology Group, *ITT* intention-to-treat, *LET* letrozole, *NE* not estimable, *OS* overall survival, *PAL* palbociclib, *PBO* placebo

Median OS was 37.5 months (95% CI 31.4–47.8) in the palbociclib plus letrozole arm and 34.5 months (27.4–42.6) in the letrozole arm (stratified hazard ratio, 0.897; 95% CI 0.623–1.294; *P* = 0.281; Fig. 1a). OS was analyzed by subgroups based on baseline characteristics such as age, ECOG performance status, disease site, prior therapy, and DFI from the end of adjuvant treatment (Fig. 1b). Nonsignificant trends in favor of palbociclib plus letrozole were observed in most subgroups; however, the number of patients in each subgroup was small, and these data should be interpreted with caution.

### Subsequent treatments

**Fig. 2:**
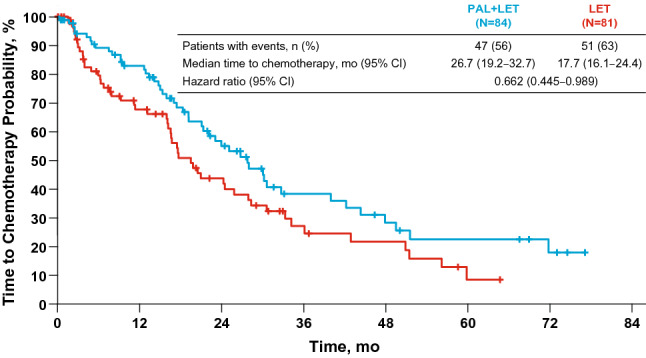
Kaplan–Meier estimated time to first use of chemotherapy postprogression. *LET* letrozole, *PAL* palbociclib

Most patients in both treatment arms received subsequent systemic therapy (83% and 89% in the palbociclib plus letrozole and letrozole arms, respectively; Table 2). In the palbociclib plus letrozole arm, 38 (47.5%), 13 (16.3%), and 15 (18.8%) patients received 1, 2, and ≥ 3 subsequent regimens of therapy, respectively. In the letrozole arm, 29 (36.7%), 11 (13.9%), and 30 (38.0%) patients received 1, 2, and ≥ 3 subsequent regimens. The median (range) number of postprogression systemic therapies was 1 (1–6) and 2 (1–9) in the palbociclib plus letrozole and letrozole arms, respectively. The most frequent subsequent systemic therapy agent was hormonal therapy (63% and 73% in the palbociclib plus letrozole and letrozole arms, respectively); the median (range) number of postprogression systemic hormonal therapies was 1 (1–3) and 1 (1–4), respectively. Fulvestrant was the most frequent subsequent hormonal therapy in both the palbociclib plus letrozole and letrozole arms (received by 34% and 43% of patients, respectively). Subsequent chemotherapy was used in 59% of patients in the palbociclib plus letrozole arm and 65% of patients in the letrozole arm. The median (range) number of chemotherapy regimens received postprogression was 1 (1–4) and 2 (1–7) in the palbociclib plus letrozole and letrozole arms, respectively. Median time from randomization to first subsequent chemotherapy was longer in the palbociclib plus letrozole arm than in the letrozole arm (26.7 and 17.7 months, respectively; Fig. 2). Twelve patients (15%) in the palbociclib plus letrozole arm and 13 patients (17%) in the letrozole arm received subsequent mTOR inhibitor (everolimus). One and 2 patients in the palbociclib plus letrozole and letrozole alone arms, respectively, received a subsequent CDK4/6 inhibitor.

**Table 2:** Summary of subsequent anticancer treatment regimens

	PAL + LET (*n* = 80)	LET (*n* = 79)
Any systemic therapy, *n* (%)	66 (83)	70 (89)
Systemic therapy agents, *n* (%)		
Antihormonal therapy	50 (63)	58 (73)
Nonsteroidal AI	14 (18)	20 (25)
Steroidal AI	21 (26)	28 (35)
Fulvestrant	27 (34)	34 (43)
Tamoxifen	11 (14)	17 (22)
Chemotherapy	47 (59)	51 (65)
Anthracyclines	15 (19)	22 (28)
Capecitabine	27 (34)	33 (42)
Gemcitabine	4 (5)	8 (10)
Taxanes	34 (43)	31 (39)
Vinorelbine	12 (15)	6 (8)
Other	19 (24)	19 (24)
mTOR inhibitor	12 (15)	13 (17)
Blinded therapy (clinical trial)	2 (3)	5 (6)
Palbociclib	1 (1)	2 (3)

*AI* aromatase inhibitor, *LET* letrozole, *mTOR* mechanistic target of rapamycin, *PAL* palbociclib

### Safety

**Fig. 3:**
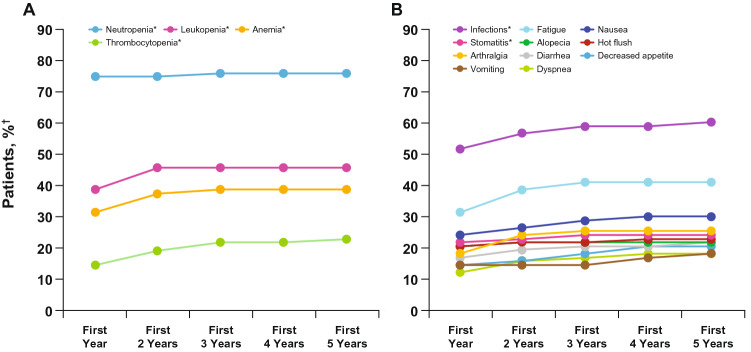
Cumulative incidence of all-causality AEs > 15% during the first 5 years of treatment with palbociclib in the as-treated set. **a** Hematologic AEs. **b** Nonhematologic AEs. *AE* adverse event. *Grouped terms were as follows: neutropenia included the preferred terms neutropenia or neutrophil count decreased; anemia included the preferred terms anemia, hematocrit decreased, or hemoglobin decreased; leukopenia included the preferred terms leukopenia or white blood cell count decreased; infections included any preferred term that is part of the System Organ Class infections and infestations; and stomatitis included the preferred terms aphthous stomatitis, cheilitis, glossitis, glossodynia, mouth ulceration, mucosal inflammation, oral pain, oropharyngeal discomfort, oropharyngeal pain, or stomatitis. †Patient percentage was calculated based on an *n* = 83 denominator

Consistent with previous reports, the most frequently reported all-causality AEs in the palbociclib plus letrozole arm were hematologic (neutropenia: any grade, 75%; grade 3 or 4, 59%; leukopenia: any grade, 43%; grade 3 or 4, 18%; Table 3). There were no reports of febrile neutropenia. The cumulative incidence of all-causality AEs reported by > 15% of patients during the first 5 years of treatment with palbociclib revealed that the incidence of AEs generally peaked within the first year and then was relatively consistent over time (Fig. 3).

**Table 3:** All-causality AEs (preferred terms) occurring in ≥ 15% of patients (AT population)

	PAL + LET (*n* = 83)					LET (*n* = 77)				
	All Grades (%)	Grade 1 (%)	Grade 2 (%)	Grade 3 (%)	Grade 4 (%)	All Grades (%)	Grade 1 (%)	Grade 2 (%)	Grade 3 (%)	Grade 4 (%)
Neutropenia	75	1	15	53	6	5	1	3	1	0
Leukopenia	43	5	21	18	0	4	1	3	0	0
Fatigue	41	16	18	5	2	23	14	8	1	0
Anemia	35	6	23	5	1	5	1	3	1	0
Nausea	30	21	7	2	0	14	8	5	1	0
Arthralgia	27	10	15	2	0	18	7	9	3	0
Hot flush	23	21	2	0	0	14	12	3	0	0
Alopecia	22	21	1	N/A	N/A	3	3	0	N/A	N/A
Diarrhea	22	8	10	4	0	12	8	4	0	0
Back pain	21	11	7	1	1	16	9	7	0	0
Decreased appetite	21	17	2	1	0	7	5	1	0	0
Thrombocytopenia	19	12	4	4	0	3	1	0	1	0
Dyspnea	18	10	5	4	0	8	4	3	1	0
Vomiting	18	13	5	0	0	4	3	0	1	0
Constipation	16	8	6	1	0	9	3	7	0	0

*AE* adverse event, *AT *as-treated, *LET* letrozole, *N/A* not applicable, *PAL *palbociclib

## Discussion

The final OS analysis showed that OS was numerically longer in the palbociclib plus letrozole arm compared with the letrozole arm, although the results did not reach statistical significance. This trend was also observed for most subgroups; however, the number of patients in each subgroup was small. Based on previous analyses and given the longer median postprogression survival observed in both treatment arms, a larger sample size would likely be needed to detect a statistically significant difference in OS in first-line ER+ breast cancer [12].

In addition, no new safety signals were observed in the current analysis; the incidence of AEs generally peaked within the first year and then remained constant for ≤ 5 years of treatment with palbociclib. The most frequently reported all-causality AEs in the palbociclib plus letrozole arm were hematologic. These data demonstrated the consistent safety profile of palbociclib in combination with letrozole with long-term use and should provide confidence that there is no cumulative toxicity. These findings are in contrast to what is commonly seen with cytotoxic chemotherapies [14]; this is especially relevant as CDK4/6 inhibitors are currently being evaluated in the early breast cancer setting [15].

Treatment with palbociclib plus letrozole prolonged the time to first use of chemotherapy compared with letrozole alone; delaying the use of cytotoxic chemotherapy can have a positive impact on patients’ quality of life [13]. While the number of patients in each treatment arm who received subsequent systemic therapy was similar, fewer patients in the palbociclib plus letrozole arm versus the letrozole alone arm received ≥ 3 subsequent systemic treatment regimens (18.8% and 38.0%, respectively). Patients in the letrozole alone arm received a median of 2 regimens of systemic therapy postprogression, driven by chemotherapy, compared with a median of 1 systemic postprogression therapy in the palbociclib plus letrozole arm. These differences could reflect the earlier time of first use of chemotherapy in the patients in the letrozole alone arm, and suggest that postprogression therapies may have impacted OS in this study. Since the results of PALOMA-1 were presented, substantial data have been generated with CDK4/6 inhibitors for the treatment of ABC, including OS data. To date, no other OS data have been presented from randomized studies of a CDK4/6 inhibitor in combination with an AI in postmenopausal women. Recently reported OS results from randomized studies showed prolonged OS with CDK4/6 inhibitors in combination with fulvestrant versus placebo plus fulvestrant [10, 16, 17]. As previously noted, a numerical but not statistically significantly longer OS was observed in the PALOMA-3 clinical trial, which evaluated the efficacy of palbociclib plus fulvestrant versus placebo plus fulvestrant for the treatment of patients with HR+/HER2− ABC whose disease had progressed on ET [10]. In MONALEESA-3, ribociclib plus fulvestrant was compared with placebo plus fulvestrant in the first- and second-line settings for postmenopausal women with HR+/HER2− ABC [16]. Ribociclib plus fulvestrant significantly prolonged OS versus placebo plus fulvestrant (hazard ratio, 0.724; 95% CI 0.568–0.924; *P* = 0.00455; median OS, not reached [NR] vs 40 months, respectively) [16]. MONARCH 2 compared abemaciclib plus fulvestrant versus placebo plus fulvestrant in women of any menopausal state (pre/perimenopausal women received ovarian suppression) with HR+/HER2− ABC whose disease had progressed on prior ET [17]. Abemaciclib plus fulvestrant prolonged OS compared with placebo plus fulvestrant (hazard ratio, 0.757; 95% CI 0.606–0.945; *P* = 0.0137; median OS, 46.7 vs 37.3 months, respectively) [17]. Of note, 33% of patients in PALOMA-3 received chemotherapy in the metastatic disease setting, whereas the MONARCH 2 and MONALEESA-3 trials did not allow prior chemotherapy for metastatic disease [10, 16, 17]. MONALEESA-7 evaluated ribociclib plus a nonsteroidal AI (NSAI) and ovarian suppression versus placebo plus ovarian suppression and an NSAI as first-line treatment in exclusively premenopausal women with HR+/HER2− ABC [18]. Ribociclib plus NSAI significantly prolonged OS versus placebo plus NSAI (hazard ratio, 0.71; 95% CI 0.54–0.95; *P* = 0.00973; median OS, NR vs 40.9 months, respectively) [18].

Since the initial readout of PALOMA-1, there have been 7 randomized phase 3 studies demonstrating the efficacy of CDK4/6 inhibition with ET versus ET alone [19–25]. These results confirm the importance of this target in ER+/HER2− breast cancer and reinforce the findings seen in this smaller, randomized study, highlighting the success of a rational development program based on preclinical observations. Limitations of the PALOMA-1 trial include its open-label design and small sample size that may limit sufficient power to detect a statistically significant difference in OS; however, the long-term safety data reported here (median duration of follow-up of > 5 years) and elsewhere [26] showed no cumulative toxicity of palbociclib plus ET and no new safety signals.

This report demonstrates a numerical increase in OS that is observed with the combination of palbociclib plus letrozole versus letrozole alone. These data, along with the published studies showing a statistically significant improvement in OS for patients receiving a CDK4/6 inhibitor in combination with various types of hormonal therapy, clearly support the use of CDK4/6 inhibitors in combination with ET as a standard of care for the treatment of patients with HR+/HER2− ABC. OS data from the phase 3 studies of NSAI in combination with CDK4/6 inhibitors in postmenopausal women, including the larger phase 3 PALOMA-2 study, are eagerly awaited.

## Data Availability

Upon request, and subject to certain criteria, conditions, and exceptions (see https://www.pfizer.com/science/clinical-trials/trial-data-and-results for more information), Pfizer will provide access to individual de-identified participant data from Pfizer-sponsored global interventional clinical studies conducted for medicines, vaccines, and medical devices (1) for indications that have been approved in the US and/or EU or (2) in programs that have been terminated (i.e., development for all indications has been discontinued). Pfizer will also consider requests for the protocol, data dictionary, and statistical analysis plan. Data may be requested from Pfizer trials 24 months after study completion. The de-identified participant data will be made available to researchers whose proposals meet the research criteria and other conditions, and for which an exception does not apply, via a secure portal. To gain access, data requestors must enter into a data access agreement with Pfizer.
